# Expression of concern: Crystalline quality assessment, photocurrent response and optical properties of reduced graphene oxide uniformly decorated zinc oxide nanoparticles based on the graphene oxide concentration

**DOI:** 10.1039/c8ra90022a

**Published:** 2018-03-14

**Authors:** Andrew Shore

**Affiliations:** Royal Society of Chemistry Thomas Graham House, Science Park, Milton Road Cambridge UK CB4 0WF advances-rsc@rsc.org

## Abstract

Expression of concern for ‘Crystalline quality assessment, photocurrent response and optical properties of reduced graphene oxide uniformly decorated zinc oxide nanoparticles based on the graphene oxide concentration’ by Majid Azarang *et al.*, *RSC Adv.*, 2015, **5**, 53117–53128.

The following article ‘Crystalline quality assessment, photocurrent response and optical properties of reduced graphene oxide uniformly decorated zinc oxide nanoparticles based on the graphene oxide concentration’ by Majid Azarang*^ab^, Ahmad Shuhaimi^a^ and M. Sookhakian^c^ has been published in *RSC Advances*.

*RSC Advances* is publishing this expression of concern in order to alert our readers to the fact that we are presently unable to confirm the accuracy of the data reported in Fig. 10, 16, and 18 and Table 4 of this *RSC Advances* paper.

We have contacted the University of Malaya to request an investigation into the validity of the published findings. We understand that an investigative process is on-going at the University of Malaya, and this notice will be updated when a final outcome is reached.

Andrew Shore

2nd March 2018

Executive Editor, *RSC Advances*

## Supplementary Material

